# Adjustment of microbial nitrogen use efficiency to carbon:nitrogen imbalances regulates soil nitrogen cycling

**DOI:** 10.1038/ncomms4694

**Published:** 2014-04-16

**Authors:** Maria Mooshammer, Wolfgang Wanek, Ieda Hämmerle, Lucia Fuchslueger, Florian Hofhansl, Anna Knoltsch, Jörg Schnecker, Mounir Takriti, Margarete Watzka, Birgit Wild, Katharina M Keiblinger, Sophie Zechmeister-Boltenstern, Andreas Richter

**Affiliations:** 1 Department of Microbiology and Ecosystem Science, Terrestrial Ecosystem Research, University of Vienna, Althanstrasse 14, 1090 Vienna, Austria; 2 Department of Forest and Soil Sciences, Institute of Soil Research, University of Natural Resources and Life Sciences, Peter-Jordan-Strasse 82, 1190 Vienna, Austria

## Abstract

Microbial nitrogen use efficiency (NUE) describes the partitioning of organic N taken up between growth and the release of inorganic N to the environment (that is, N mineralization), and is thus central to our understanding of N cycling. Here we report empirical evidence that microbial decomposer communities in soil and plant litter regulate their NUE. We find that microbes retain most immobilized organic N (high NUE), when they are N limited, resulting in low N mineralization. However, when the metabolic control of microbial decomposers switches from N to C limitation, they release an increasing fraction of organic N as ammonium (low NUE). We conclude that the regulation of NUE is an essential strategy of microbial communities to cope with resource imbalances, independent of the regulation of microbial carbon use efficiency, with significant effects on terrestrial N cycling.

## Abstract (web summary)

Nitrogen availability in soils is predominantly controlled by microorganisms, yet our understanding of their organic nitrogen use is limited. Mooshammer *et al.* show that microbial nitrogen use efficiency is dependent on resource stoichiometry and substrate type.

## Graphical abstract

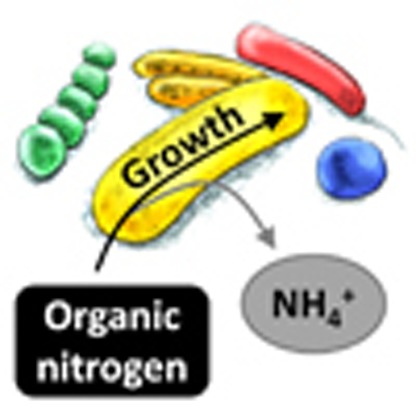

In the soil N cycle, the rate-limiting step of microbial decomposition of organic matter is the depolymerization of proteins to oligopeptides and amino acids by extracellular proteases, rather than the subsequent mineralization of amino acids to ammonium^1^,^2^,^3^. The products of the depolymerization process can be directly and rapidly utilized by microbes as both energy and nutrient sources^4^,^5^. However, most studies on soil N cycling have focused on N mineralization, rather than on the partitioning of organic N between incorporation into microbial biomass and release as ammonium. A thorough understanding of microbial nitrogen use will thus strongly improve our knowledge about how heterotrophic microbes control soil inorganic N availability, and thereby regulate ecosystem functions, such as plant productivity. Microbial nitrogen use efficiency (NUE) reflects the partitioning of organic N taken up (*U*_N_), between N incorporation into microbial biomass (growth; *G*_N_) and N recycling to the environment as inorganic N (mainly as ammonium; N mineralization, *M*_N_; see also Supplementary Fig. 1; Supplementary Note 1 for integration of NUE in the microbial N balance), which can be formulated as:


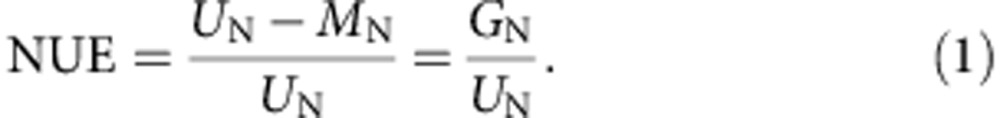


High NUE expresses efficient N sequestration of the N taken up into microbial biomass and the concomitant release of only a small fraction of that N back to the environment as inorganic N (that is, low N mineralization). In contrast, low NUE indicates that less N is converted to biomass, whereas a relatively large fraction of the organic N taken up is released as ammonium (that is, high N mineralization).

While microbial NUE has not been studied so far, microbial carbon use efficiency (CUE; that is, the efficiency by which organisms convert organic C into biomass C) has been the focus of many studies in soil biogeochemistry, and has been established as a main factor determining microbial growth, nutrient immobilization and ultimately soil C sequestration^6^,^7^. Microbial C metabolism is a highly regulated interplay between anabolic (providing the biochemical basis for growth) and catabolic processes (for example, respiration)^8^. The metabolic control of microbial anabolism and catabolism therefore effectively drives changes in microbial CUE. Empirical as well as modelling studies demonstrated stoichiometric and environmental controls on microbial CUE^7^,^9^,^10^,^11^,^12^, and illustrated its importance for a range of different biogeochemical processes in marine and terrestrial ecosystems^6^,^11^,^13^. Although NUE has been taken into account in some theoretical and conceptual models of organic matter decomposition^14^,^15^, its plasticity and regulation have not yet been empirically tested, despite the critical importance of N as a limiting nutrient for terrestrial primary productivity^16^. However, similar to microbial C metabolism, N metabolism is central to the physiological functioning of microbes and therefore has been shown to be strongly controlled^8^,^17^. The balance between anabolic processes (such as protein biosynthesis and growth) and catabolic processes (that eventually lead to the exudation of catabolic products in excess of microbial N demand) in microbes is highly regulated; we can thus predict microbial NUE to be flexible and regulated, similar to CUE. Moreover, NUE is expected to follow its own controls, although controls of microbial CUE and NUE may overlap due to cross-talk between microbial C and N metabolism^8^,^18^, and given that elements other than C or N (for example, P) can become limiting for microbial growth, in which case both CUE and NUE must be regulated independently to adjust biomass to resource stoichiometry. Although microbial CUE and NUE are expected to be flexible and may have divergent controls, the mass balance equation


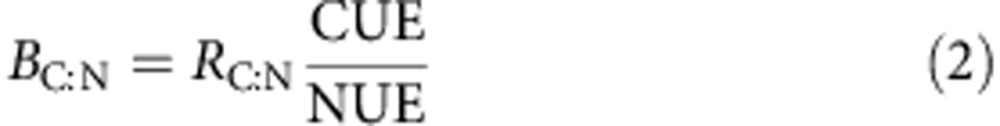


predicts that microbial CUE, NUE and biomass C:N (*B*_C:N_) are interrelated and dependent on resource C:N (*R*_C:N_), and therefore in strictly homeostatic microbes these parameters are not fully independent.

Ecological stoichiometry uses elemental ratios and the concept of stoichiometric invariance (homeostasis) to predict nutrient retention and biomass production, from subcellular to ecosystem scales^14^. The theory of ecological stoichiometry suggests that at low substrate C:N ratios (N-sufficient conditions), strictly homeostatic organisms have low NUE but high CUE^14^. In contrast, at high substrate C:N ratios (N-deficiency) they are expected to lower their CUE while increasing their NUE. A key approach for understanding nutrient limitation in decomposers is therefore the threshold elemental ratio (TER) that expresses the elemental ratio at which metabolic control of an ecological system switches from C limitation to nutrient (N or P) limitation^19^,^20^,^21^. TER_C:N_ denotes the threshold of the resource C:N below which N will be in excess in relation to the organism’s N requirements, and consumers therefore become C or energy limited, and above which N will limit organismic growth. By integrating the regulation of NUE and CUE by resource C:N into the TER concept, we expect microbial NUE to decrease below the TER_C:N_ when N is in excess, and C is the limiting element (Fig. 1). In contrast, above this threshold microbial communities are expected to be N limited, while C is in excess, and NUE should consequently reach a maximum accompanied by down-regulation of CUE. Non-homeostatic decomposers, in turn, may alter their cellular composition to limit the stoichiometric imbalance to their resource^14^. However, elemental imbalances are likely to emerge, even when microbial biomass C:N varies, because the variability in their cellular composition is limited by physiological bounds, and adjustments in CUE and NUE are therefore expected to occur.

Towards a more mechanistic understanding of the soil N cycle, we here explore the ability of terrestrial decomposer communities to adjust their N metabolism (NUE) to N availability. We analyse microbial NUE by measuring gross fluxes of organic N uptake and ammonium excretion (N mineralization) in soils and decomposing plant litter varying in their C:N ratios. By applying these simplifying assumptions of ecological stoichiometry^14^, we are able to capture the metabolic flexibility of microbial communities across different substrate types and qualities. Our results show that microbial NUE varies considerably among microbial communities in different substrate types, and that the C:N imbalance between resource and microbial biomass is also compensated by adjustments in microbial NUE and not solely by flexibility in microbial CUE and biomass C:N.

## Results

### The magnitude of microbial NUE

Microbial NUE in different substrate types (that is, decomposing plant litter, mineral and organic soil horizons) varied between 0.15 and 1 (Fig. 2a). Microbial NUE in soil exhibited greater variation (coefficient of variance, CV=30.7% and 17.8% for mineral and organic soil horizon, respectively) than in plant litter (CV=8.9%). Microbial NUE was lower in mineral soil horizons (0.70±0.21 s.d.; *n*=36) compared with organic soil horizons (0.83±0.15 s.d.; *n*=19) and plant litter (0.89±0.08 s.d.; *n*=38), although this difference was significant only between the mineral soil horizon and plant litter (Kruskal–Wallis test followed by Dunn’s test, *H*(2)=18.2, *P*<0.001; litter versus mineral, *Q*=4.26, *P*<0.05; litter versus organic, *Q*=1.49, *P*>0.05; organic versus mineral, *Q*=2.02, *P*>0.05).

To meet their N demand, microbial communities also take up inorganic N that becomes available through N mineralization and nitrification. In our study, ammonium concentrations were on average 14.1 (±7.1 s.d.) in plant litter, 18.9 (±39.8 s.d.) in organic and 5.3 μg N g^−1^ dry weight (±6.7 s.d.) in mineral soil horizons. Average nitrate concentrations ranged between 4.1 (±2.1 s.d.) in plant litter, 1.4 (±3.0 s.d.) in organic and 1.0 μg N g^−1^ dry weight (±0.7 s.d.) in mineral soil horizons. For plant litter samples, we also measured gross nitrification and nitrate consumption rates in addition to gross ammonification and ammonium consumption rates in order to include inorganic N assimilation in microbial NUE (NUE_+inorg_). When accounting for inorganic N assimilation, we found an average NUE_+inorg_ of 0.81 (±0.09 s.d.). Microbial NUE and NUE_+inorg_ were strongly correlated with a slope close to 1 (*NUE*_+inorg_=1.012 × *NUE*−0.089; *R*^2^=0.801; *P*<0.001) showing that inorganic N assimilation contributed proportionally across all plant litter samples.

### Adjustment of microbial NUE to different resource C:N ratios

The C:N ratios of plant litter, organic and mineral soil horizons ranged from 9 to 62 (on a mass basis). The soil samples covered a representative range of soil C:N (9–38) comparable to major terrestrial biomes (9–35, including 95% confidence interval)^22^. Soil microbial communities commonly exhibit a high degree of resource homeostasis, that is, relatively constant biomass stoichiometry independent of resource stoichiometry^14^. In this study, microbial biomass C:N ranged between 4 and 25. Average microbial biomass C:N in plant litter, organic and mineral soil horizons was 9 (CV=16.2%; *n*=38), 7 (CV=13.3%; *n*=13) and 9 (CV=65.9%; *n*=20), respectively. When plotting the logarithm of microbial biomass C:N as a function of the logarithm of resource C:N, which represents the dependency of the stoichiometry of the microbial decomposer community on resource stoichiometry, we found a weak positive relationship between the C:N ratios of microbes and resources (Fig. 3; slope=0.14; *P*<0.05). The slope being below 0.25 indicates C:N homeostasis (though not a strict homeostasis) of the microbial decomposer community across the substrate types studied here^14^,^23^. We found no positive relationship between the logarithm of total dissolved C:N and microbial biomass C:N (slope=0.05, *P*=0.309; data not shown).

**Figure 1:**
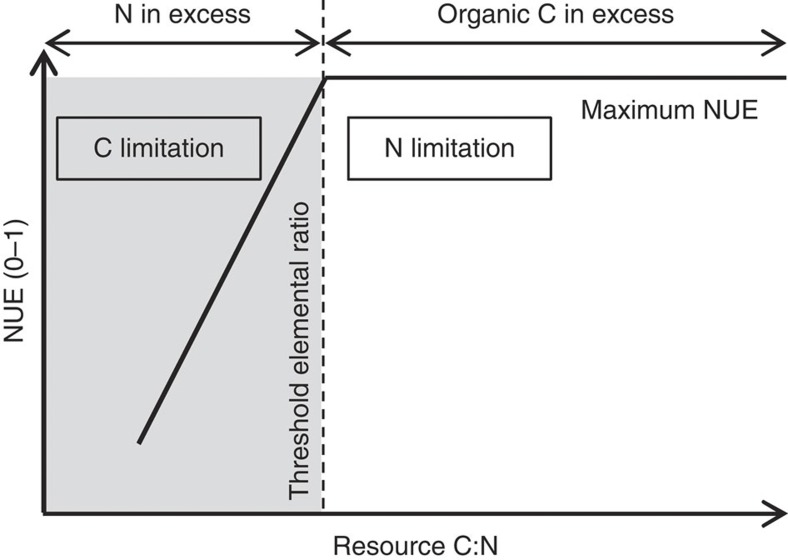
Conceptual diagram illustrating the regulation of NUE in a homeostatic heterotrophic microbial community. The threshold elemental ratio denotes the threshold of the resource C:N below which N will be in excess in relation to the demand of the microbial community (C limitation). Excess N is expected to be released causing a reduction in microbial NUE. In contrast, above this threshold the microbial community is expected to be limited by N. Consequently, microbial NUE reaches a maximum value.

**Figure 2:**
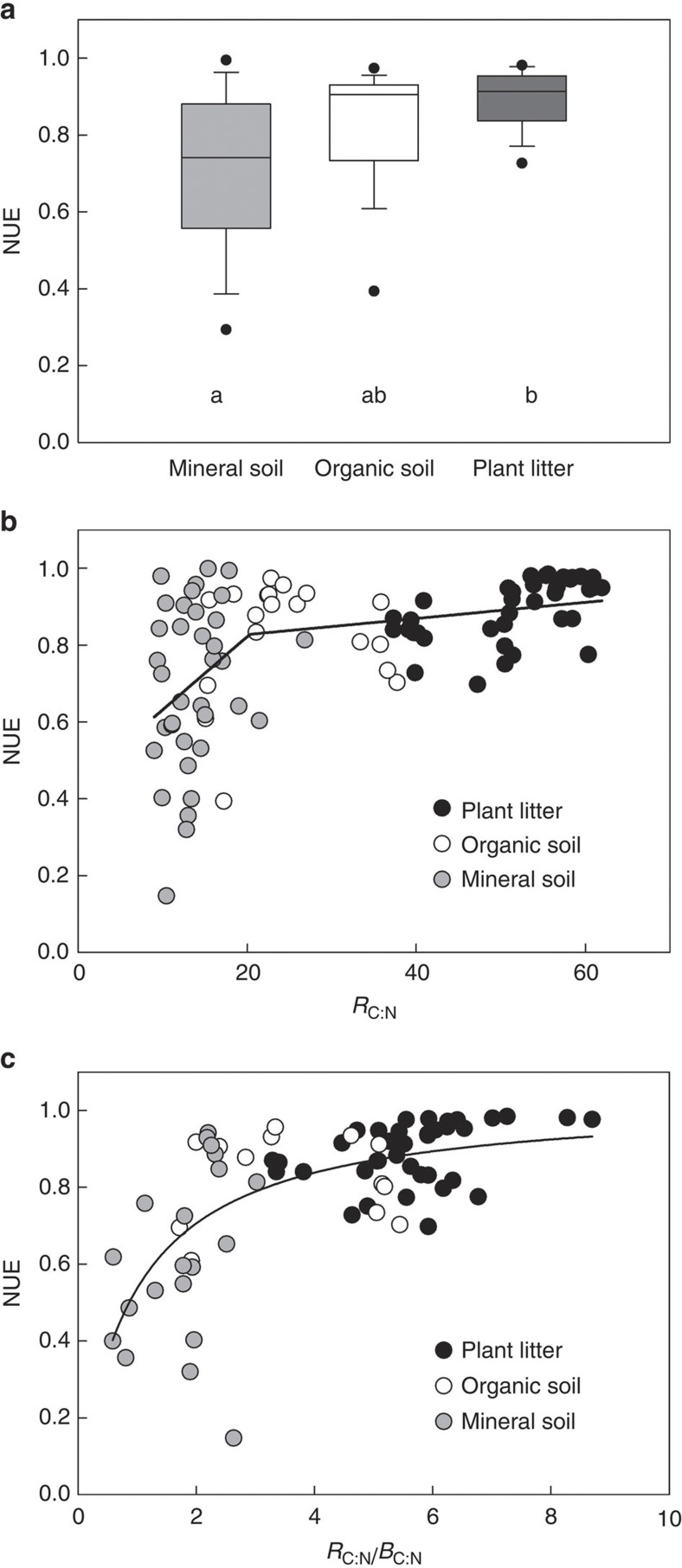
Microbial NUE. (**a**) Box plots showing microbial NUE in mineral (*n*=36), organic soil horizons (*n*=19) and in decomposing plant litter (*n*=38). Different letters indicate significant differences in NUE between substrate types (Kruskal–Wallis test followed by Dunn’s test). The box plots show the medians (solid line within boxes), 25th and 75th percentiles as vertical bars, 10th and 90th percentiles as error bars and 5th and 95th percentiles as circles. (**b**) Relationship between resource C:N (*R*_C:N_; mass basis) and microbial NUE for litter, organic and mineral soil horizons. Solid lines are linear regression lines of the piece-wise regression model (*R*^2^=0.301; *F*_3,89_=12.74; *P*<0.001; *n*=93). A significant break point was found at a resource C:N of 20 with a corresponding value of 0.83 for microbial NUE. (**c**) Relationship between C:N imbalance and microbial NUE for litter, organic and mineral soil horizons. The stoichiometric imbalance between microbial decomposers and their resource can be represented by resource C:N (*R*_C:N_) normalized to microbial biomass C:N (*B*_C:N_). The relationship was best described by a saturating nonlinear regression model as follows: *NUE*=1.03 × (*R*_C:N_/*B*_C:N_)/[0.92+(*R*_C:N_/*B*_C:N_)] (*R*^2^=0.431; *F*_1,69_=52.27; *P*<0.001; *n*=71).

**Figure 3:**
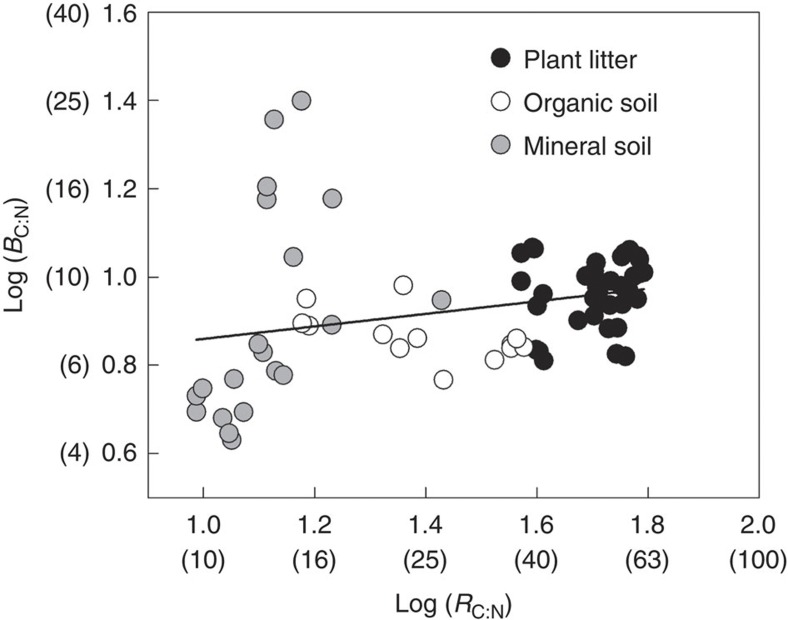
Homeostasis in microbial decomposer communities. Stoichiometric homeostasis can be described by a linear relationship between the logarithm of resource C:N (*R*_C:N_) and microbial biomass C:N (*B*_C:N_) (slope=0.14; *P*<0.05; *n*=71). Non-log-transformed values for *R*_C:N_ and *B*_C:N_ are given in parentheses.

Figure 2b shows microbial NUE plotted against resource C:N that displays a partitioning of the NUE relationship into two intervals, that is, at low resource C:N microbial NUE increased sharply, whereas at high resource C:N the increase of microbial NUE was less pronounced. The relationship between resource C:N and microbial NUE was first tested by a classification and regression tree (CART) analysis. The optimal tree contained one node at a resource C:N of 15, with a cross-validation error of 0.866 and *R*^2^ equivalent of 0.31. Following the CART analysis, which showed one split in the data set, and according to our theory of one threshold (Fig. 1), we performed a piece-wise (two-segmented) regression analysis of microbial NUE against resource C:N that revealed a break point at a resource C:N of 20 (±4.6 s.e.), with a corresponding value of 0.83 for microbial NUE (*n*=93; *R*^2^=0.301; *P*<0.001; Fig. 2b).

The stoichiometric imbalance between microbial decomposers and their resource can be calculated as the resource C:N normalized to microbial biomass C:N. Given that biomass stoichiometry reflects the stoichiometric requirements of microbial decomposers, an increasing stoichiometric imbalance indicates increasing microbial N limitation (or decreasing C limitation). We found that microbial NUE increased nonlinearly with increasing C:N imbalance from mineral soil horizons to plant litter (Fig. 2c). This relationship was best described by a saturating nonlinear regression model (*R*^2^=0.431; *P*<0.001), without a break point in the relationship.

Following the theoretical consideration mentioned above that at high resource C:N (above TER_C:N_) microbial NUE reaches a maximum and remains invariant due to N limitation of decomposer communities while CUE is regulated to cope with resource imbalances (Fig. 1), we also analysed decomposing plant litter independently of soil samples. We found that above the TER_C:N_, microbial NUE still increased with increasing litter C:N (*NUE*=0.601+0.0056 × *R*_C:N_; *R*^2^=0.297; *F*_1,36_=15.21; *P*<0.001; Supplementary Fig. 2), although less rapidly than below the TER_C:N_.

## Discussion

Our results demonstrate that microbial NUE and consequently N mineralization relative to organic N uptake was not constant and varied considerably among microbial communities decomposing different substrate types (Fig. 2a). Notably, microbes in organic soil horizons and plant litter were the most efficient in sequestering organic N into their biomass (83% and 89%, respectively). Such high microbial NUE values also denote a reduced potential for soil N losses by providing less substrates for nitrification and consequent losses through gaseous N forms (for example, denitrification and nitrifier denitrification) and nitrate leaching. It has indeed been shown that N-limited ecosystems have comparatively lower N losses (export of nitrate and gaseous N relative to dissolved organic N)^24^. Furthermore, high levels of microbial NUE also indicate that microbes strongly contribute to N sequestration in soil organic matter, emphasizing the role of microbial biomass as a major sink of N in soils^25^. The continuous turnover of N, through the repetitive process of microbial growth and death, also constitutes an important mechanism of N conservation in ecosystems. This is supported by the fact that soil organic matter, particularly soil organic N, is predominantly of microbial origin, constituting up to 80% of soil organic N^26^. Our results, that is, higher NUE in microorganisms decomposing organic matter with a low N content in relation to C (that is, plant litter and organic soil horizons), demonstrates that microbial communities can efficiently capture N in N-limited ecosystems.

Microbial communities inhabiting different environments are exposed to resources with different elemental and chemical compositions, as well as different nutrient availabilities. Globally, plant litter exhibits considerably wider C:N:P ratios (a mass ratio of 1,166:20:1)^27^,^28^ in relation to that of soil organic matter (111:8:1) and soil microorganisms (16:3:1)^22^,^29^. The resultant stoichiometric imbalance between resources and microbial decomposers, however, poses significant stoichiometric constraints on the physiology of microorganisms, and consequently on microbially mediated ecosystem processes^14^. Despite the fact that microbial growth in soils can be either energy (C) or nutrient (N) limited or both, declining C:N ratios from litter to soil indicate decreasing C availability (increasing C limitation) in relation to N, and therefore decreasing N limitation. At high resource C:N, decomposers conserve N and liberate the excess C via overflow respiration only until reaching a certain C:N threshold at which microbial growth limitation switches from N to C limitation, and excess N is released via N mineralization. The threshold at a resource C:N of 20 found in this study is expected to approximate TER_C:N_ (Fig. 2b). TER_C:N_ has been considered to range between 20 and 25 based on empirical studies that measured the critical transition from net N immobilization to net N mineralization in organic matter decomposition^30^. The present results demonstrate that stoichiometric constraints on microbial physiology lead to a reduction in microbial NUE at low resource C:N (N-sufficient substrate). In our analysis, microbial NUE was consistently high above the TER_C:N_, which is expected to be accompanied by a decrease in microbial CUE as shown for soils where microbial CUE decreased with increasing C:N^31^. Identifying and estimating critical thresholds underlying changes in fundamental microbial processes is thus crucial in order to understand and model broad-scale ecosystem processes, such as soil C sequestration and N cycling.

In decomposing litter, we found that microbes regulated their NUE with increasing resource C:N even above the TER_C:N_ (where N was limiting and NUE should be at the maximum) (Supplementary Fig. 2). This is important since it demonstrates that decomposer communities not only regulate their NUE at low resource C:N when N is in excess, but also at high resource C:N at which maintenance of biomass elemental homeostasis is generally thought to be achieved solely by regulating microbial CUE. Previous modelling studies indicated that microbial decomposers may adapt to low N (high C) conditions by reducing their CUE^11^,^12^, overlooking that NUE may also vary. Although it has been suggested for N-poor substrates that a less efficient microbial use of C (lower CUE) will be accompanied by a more efficient microbial use of N (higher NUE)^11^, our results provide the first experimental evidence for this hypothesis. Beyond the carbon-centric view of microbial C and nutrient cycling, which focuses on the effect of resource stoichiometry on microbial CUE and related biogeochemical processes^11^,^12^, we demonstrate here that the stoichiometric C:N imbalance between resource and microbial biomass may not only be compensated by an adjustment of CUE (and smaller adjustments in biomass stoichiometry), but also by changes in the efficiency of organic N assimilation (NUE).

Given that biomass stoichiometry is the basis for the nutrient requirements of microbial decomposers, an increasing stoichiometric imbalance (here calculated as resource C:N over biomass C:N) indicates increasing microbial N limitation. Microbial NUE increased nonlinearly with increasing C:N imbalance, explaining 43% of the variation in microbial NUE across all substrate types tested (Fig. 2c). This strong relationship shows that NUE of terrestrial microbial communities is strongly regulated by the stoichiometric imbalance between the resource being decomposed and the physiological nutrient demand of microbes across different resource qualities.

Microbial NUE and CUE are community characteristics and as such represent the integration of the activity of a diverse assemblage of microbes, and reflect the complex interplay between them and the variety of resources available. In this study, the TER_C:N_ was ~3.8 times higher than microbial biomass C:N (5.4; calculated from the regression between C:N imbalance and microbial NUE using a value for NUE of 0.83 at a TER_C:N_ of 20). This suggests that decomposer communities, when switching from energy (C) to N limitation, respired ~3.8 times more C than that used for growth only (that is, biomass production). Microbial CUE strongly depends on substrate quality and has been shown to be 0.55 on average in soils^7^. However, it has been recently suggested that this average terrestrial CUE represents a gross overestimate owing to methodological biases, and that terrestrial CUE converges towards 0.3 based on kinetic and metabolic considerations^32^. The TER_C:N_ can be represented by the product of the ratio of physiological N and C assimilation efficiency (microbial NUE and CUE) and biomass elemental composition (*B*_C:N_)^14^:


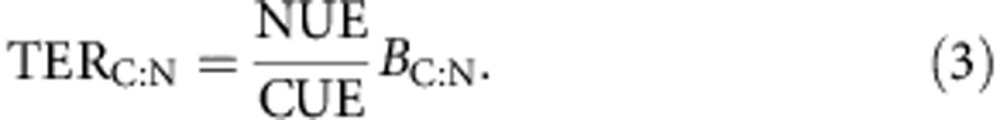


According to this equation, microbial CUE in our study would be 0.22 at TER_C:N_, which is close to the above mentioned CUE estimate of 0.3.

Besides controls through resource C:N imbalances, which inversely affect CUE and NUE (although not necessarily to the same extent), there are several other factors that can modulate NUE and CUE. First, in the case of microbes being limited by an element other than C or N, both CUE and NUE must be lowered to ensure microbial homeostasis. Second, different assemblages and amounts of extracellular enzymes are needed to deconstruct different substrates. Enzyme production involves both C and N investment, but more N relative to C is necessary for enzyme than for biomass production (C:N of enzymes ≈3)^33^, potentially decreasing NUE more than CUE. Third, a large fraction of microorganisms in natural environments, especially in soils, are metabolically inactive^34^,^35^. Costs associated with microbial dormancy and survival in the dormant state possibly decrease CUE of microbial communities through low but steady maintenance respiration in the absence of C uptake, but would not affect NUE. Fourth, physiological responses of microorganisms to stress may also lead to shifts in the allocation and fate of C and N, depending on the prevailing osmolyte produced, for example, amino acids versus polyols^36^. There are potentially more such factors that differentially affect CUE and NUE, and a better knowledge of these factors is strongly needed to gain a thorough mechanistic understanding of soil C and N cycling by microorganisms.

We conclude that the regulation of NUE is an essential strategy of microbial communities to cope with resource variability and elemental imbalances, and therefore to maintain microbial elemental homeostasis. We provide evidence that the C:N imbalance between resources and microbial biomass is also compensated by adaptations in NUE and not solely by CUE, as usually assumed. Such adaptations in microbial NUE potentially have a critical impact on soil N cycling, such as the modulation of N losses (for example, N conservation in N-limited ecosystems). Microbial NUE is a fundamental parameter to understand and predict ecosystem N dynamics and N sequestration, particularly in response to environmental changes. The observed pattern in microbial NUE provides a first framework to incorporate variable NUE of microbial communities in process-based biogeochemical models.

## Methods

### Experimental design

To relate microbial NUE to resource C:N, we analysed soil and plant litter samples (*n*=93) in order to cover a wide range of C:N ratios. Soil samples were collected from two tundra sites in August 2010 (Greenland; 74°29′ N, 20°32′ W; Russia; 68°45′ N, 161°36′ E; *n*=28)^37^, two boreal forest sites in August 2012 (Russia, *Picea sp.* dominated forest, 63°17′ N, 74°33′ E; *Abies sp.* dominated forest, 58°18′N, 68°35′ E; *n*=19) and a temperate (subalpine) grassland site in May 2012 (Austria, 47°07′ N, 11°18′ E; *n*=8). We sampled topsoil (organic horizon) and subsoil (mineral horizon) from replicated soil pits, except grassland soil samples, where only the upper mineral horizon was sampled. Living plant roots were removed from the soil, and the soil was homogenized or sieved to 2 mm where appropriate. Moreover, we carried out a litter decomposition experiment in order to analyse microbial NUE at different litter C:N ratios under controlled conditions. For this purpose, beech litter (*Fagus sylvatica* L.) varying in elemental stoichiometry (C:N:P) but not in litter C chemistry^38^ was collected at four different locations in Austria in October 2007: Achenkirch (AK), Ossiach (OS), Klausenleopoldsdorf (KL) and Schottenwald (SW) referred to as ‘litter types’. Site characteristics as well as the litter treatment are described in ref. 39. In short, the collected litter was dried at 40 °C for 48 h, finely chopped (1–20 mm) and sterilized by gamma-ray treatment. In order to obtain the same initial microbial community for all litter types, the sterilized litter was inoculated with a O-horizon:litter mixture (1:1 (w:w)) from Klausenleopoldsdorf collected in December 2007. Of each inoculated litter type 60 g were placed in mesocosms constructed from polyvinyl chloride (PVC) tubes (height 10 cm, diameter 12.5 cm) and kept at 15 °C throughout the experiment. Litter water content was maintained at 60% fresh weight by adding autoclaved tap water weekly. Litter decomposition was followed over a period of 6 months with two samplings (3 and 6 months after the start of the experiment).

### Analysis of organic matter and microbial biomass

Dry mass of soils and plant litter was determined by drying at 80 °C for 48 h. Total C and N content of the litter samples were determined after grinding with a ball mill with an elemental analyzer (Leco CN2000, Leco Corp. St Joseph, MI, USA). Total C and N content of soil samples were determined using a continuous-flow isotope ratio mass spectrometer (IRMS) consisting of an elemental analyzer (EA 1110, CE Instruments, Milan, Italy) coupled via a ConFlo III interface (Finnigan MAT, Bremen, Germany) to the IRMS (DeltaPLUS, Finnigan MAT), except tundra soil samples from Russia, which were measured by Isoprime elemental analyzer-isotope ratio mass spectrometer (EA-IRMS) system coupled to an Agilent Technology 7890A GC (Agilent Technologies, Santa Clara, CA). Tundra soil samples contained traces of carbonate and were acidified in HCl atmosphere and neutralized over NaOH before EA-IRMS analysis. Microbial biomass C and N were determined by CHCl_3_ fumigation-extraction^40^ and analysed with a TOC-V_CPH/CPN_/TNM-1 analyzer (Shimadzu, Japan).

Microbial biomass C and N were not determined for tundra soil samples and were below detection limit in subsoil mineral horizons (Bg and E horizons) from the boreal forest soil samples. We did not apply correction factors (k_EC_ and k_EN_) for incomplete extraction of microbial biomass C and N by the CHCl_3_ fumigation-extraction method, as these factors have not been tested explicitly for plant litter. The elemental stoichiometry (C:N) of soil, plant litter and microbial biomass are here expressed as mass ratios.

Ammonium and nitrate were quantified by colorimetric methods, ammonium via a modified Berthelot reaction and nitrate by a VCl_3_-Gries reaction as published recently^41^.

### Microbial NUE

Microbial NUE is the fraction of consumed organic N (free amino acids) that is not released as ammonium and therefore incorporated into microbial biomass. NUE is calculated as follows:


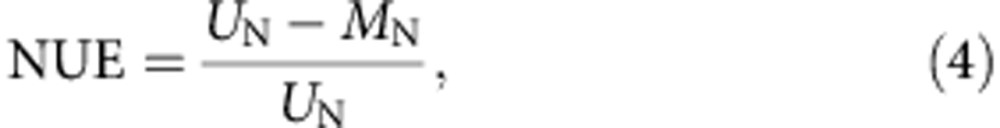


where *U*_N_ is microbial uptake of organic N and *M*_N_ is N mineralization. NUE is dimensionless and can range between 0 and 1. Proteinaceous substances are the major N-containing compounds in litter and soil^42^,^43^,^44^, which become bioavailable through enzymatic depolymerization to small peptides and free amino acids by extracellular proteases and peptidases. Free amino acids represent a small but highly dynamic pool of organic N in litter^3^,^39^ and soils, with a half-life of minutes to hours^5^,^45^. In decomposing litter, free amino-acid uptake exceeded ammonium and nitrate uptake by microbes by a factor of >8, which points at the pivotal role of free amino acids as the major form of N to meet the microbial N demand^39^. Therefore, we measured gross rates of amino-acid consumption to determine *U*_N_. *M*_N_ was determined by the gross N mineralization rate (that is, gross production of NH_4_^+^). The term *U*_N_−*M*_N_ represents the fraction of organic N incorporated into microbial biomass. Gross amino-acid consumption rates were determined using the isotope pool dilution technique according to ref. 39. Litter and soil samples were slightly differently treated owing to difference in physical properties and in total free amino-acid pool size. We added 4–40 μg (litter) or 1.25–5 μg (soil) of uniformly-^15^N-labelled amino-acid mix (98 atom% ^15^N, 20 amino acids) to 2 g (litter) or 1–4 g (soil) fresh material in triplicates (litter) or duplicates (soil). The samples were incubated at 15 °C (litter, temperate grassland soil and boreal forest soil) or 7 °C (tundra soil) and the assays were terminated after 2, 10 and 20 min (litter) or 10 and 30 min (soil) by adding 14 ml (litter) or 19.5 ml (soil) 10 mM CaSO_4_ containing formaldehyde (end concentration 3.7%), which effectively inhibited protease activity without lysing microbial cells. Concentrations and ^15^N:^14^N ratios of individual amino acids were determined by compound-specific isotope analysis via Gas chromatography–mass spectrometry. To quantify gross N mineralization and nitrification, quadruplicate samples of moist soil (2–4 g) were treated by addition of 2.5 ml 0.1 mM NH_4_Cl or KNO_3_ (10 atom% ^15^N) and litter samples (1.5 g) by addition of 0.5–1 ml 0.125 mM NH_4_Cl or KNO_3_ (10 atom% ^15^N), respectively. Assays were terminated after 4 and 24 h by extraction with 13 ml or 12.5 ml 2 M KCl for soil and litter samples, respectively. The samples were shaken (soil samples, 30 min; litter samples, 60 min) and filtered through ash-free cellulose filter paper. To stabilize the soil extracts, 20 μl of 5 mM phenylmercuric acetate were added and samples were frozen until further processing. Ammonium and nitrate were isolated by microdiffusion with sequential addition of MgO and Devarda’s alloy^46^. Acid traps were dried and analysed for N content and atom% ^15^N by a continuous-flow IRMS consisting of an elemental analyzer (EA 1110, CE Instruments) coupled via a ConFlo III interface (Finnigan MAT, Thermo Fisher) to the IRMS (DeltaPLUS, Finnigan MAT, Thermo Fisher).

### Data and statistical analyses

In order to analyse the effect of substrate type (decomposing plant litter, mineral and organic soil) on microbial NUE, we used the Kruskal–Wallis test (assumptions for parametric procedure were not met) followed by a non-parametric multiple comparison test (Dunn’s test), which compares the difference in the sum of ranks between the groups with the expected average difference. Across all substrate types, the relationship between NUE and resource C:N was first analysed using CART analysis, which is inherently non-parametric. In other words, no assumptions are made regarding the underlying distribution of values of the predictor variables. Optimal tree size was determined using the 1-SE rule^47^. Following the CART analysis, we used piece-wise linear regression analysis to more thoroughly investigate the relationship that emerged in the CART analysis. In piece-wise regression models that are effective in modelling abrupt thresholds, two or more lines are joined at unknown point(s), called ‘break point(s)’, representing threshold(s)^48^. Assumptions for the piece-wise regression model (normal distribution and homoscedasticity of residuals) were met. We used a saturating nonlinear model to describe the relationship between NUE and C:N imbalance. For the litter decomposition experiment, simple linear regression analyses were used to relate NUE to resource C:N. The Kruskal–Wallis test, Dunn’s test and piece-wise regression analysis were performed in Sigmaplot 11.0 (Systat Software Inc., San Jose, CA, USA; www.sigmaplot.com), linear and nonlinear regression analyses in Statgraphics 5.0 (Statistical Graphics Inc., Rockville, MD, USA; www.statgraphics.com). CART analysis was performed with the rpart library in R (version 2.12.1)^49^,^50^.

## Author contributions

W.W., A.R. and S.Z.-B. designed research. M.M., I.H., J.S., B.W., M.W., L.F., A.K., M.T. and K.M.K. performed the experiments. M.M., F.H. and W.W. analysed the data. M.M., W.W. and A.R. wrote the manuscript.

## Additional information

**How to cite this article:** Mooshammer, M. *et al.* Adjustment of microbial nitrogen use efficiency to carbon:nitrogen imbalances regulates soil nitrogen cycling. *Nat. Commun.* 5:3694 doi: 10.1038/ncomms4694 (2014).

## Supplementary Material

Supplementary Information Supplementary Figures 1-2, Supplementary Note 1 and Supplementary References
