# Supplementary material for: Adjustment of microbial nitrogen use efficiency to carbon:nitrogen imbalances regulates soil nitrogen cycling
Source: Nat Commun. 2014 Apr 16;5:3694. doi: 10.1038/ncomms4694 (PMC3997803; doi:10.1038/ncomms4694)
Supplement: Supplementary Information — Supplementary Figures 1-2, Supplementary Note 1 and Supplementary References [file ncomms4694-s1.pdf]

SUPPLEMENTARY FIGURES

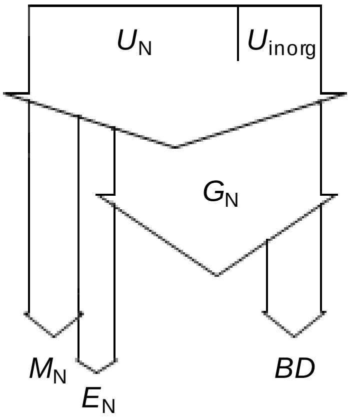

Supplementary Figure 1. Schematic illustration of the microbial N balance. Arrows represent N fluxes (U_N, microbial organic N uptake; U_{inorg}; microbial inorganic N uptake; G_N, microbial net growth; M_N, release of excess N; E_N, excretion of metabolites including extracellular enzymes; *BD*, microbial mortality).

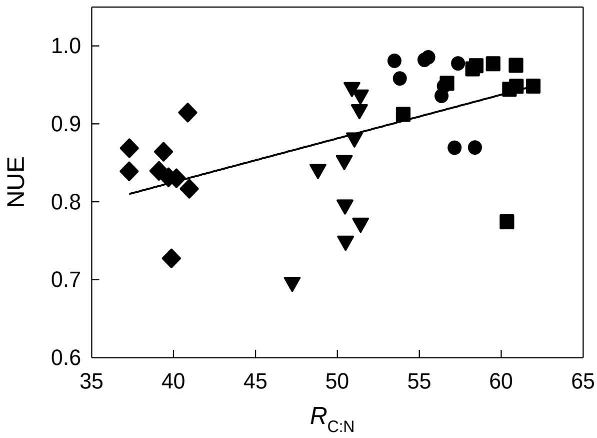

Supplementary Figure 2. Relationship between resource C:N ($R_{C:N}$) and microbial NUE in decomposing plant litter. Different symbols indicate four beech litter types with different C:N ratios. The solid line is a fitted simple linear regression model (NUE=0.601+0.0056×$R_{C:N}$; R^2=0.297, $F_{1,36}$=15.21; P<0.001; n=38).

SUPPLEMENTARY NOTE 1

Integration of NUE in the microbial N balance

Net changes in microbial N (B_N) result from the difference between inward and outward flows from the microbial N pool (see Supplementary Fig. 1 for schematic illustration) and can be described as

$$\frac{\mathrm{d}B_{\mathrm{N}}(t)}{\mathrm{d}t} = U_{\mathrm{N}} + U_{\mathrm{inorg}} - M_{\mathrm{N}} - E_{\mathrm{N}} - BD = G_{\mathrm{N}} - BD, \quad (1)$$

where U_N is microbial organic N uptake, U_{inorg} is microbial inorganic N uptake, M_N is the release of inorganic N (gross N mineralization), E_N represents the excretion of metabolites (particularly extracellular enzymes), BD is microbial mortality and G_N is microbial net growth (adapted from the mass balance of microbial biomass C in[1]). Organic and inorganic N taken up by microbes is used for microbial growth (sequestration in biomass) and a small fraction is devoted to extracellular enzyme production. Organic N in excess to microbial requirement can be mineralized (excreted) providing inorganic N (NH_4^+) for uptake by other organisms. In the short-term, the difference between N uptake (U_N+U_{inorg}) and N mineralization plus excretion of metabolites and extracellular enzymes (M_N+E_N) represents the fraction of N incorporated in biomass (G_N). Microbial N investment into extracellular enzymes is expected to be less than 5% (e.g., bacteria were shown to use 1–5% of N assimilated for extracellular enzyme production[2]). Given that E_N is <5% of U_N+U_{inorg}, the term U_N+U_{inorg}−M_N can be replaced by G_N, which represents net microbial growth.

Microbial NUE can then be defined as the ratio of N used for growth over N uptake. Based on this definition and on the mass balance of Supplementary equation 1, NUE is given as

$$NUE = \frac{G_{\mathrm{N}}}{U_{\mathrm{N}}+U_{\mathrm{inorg}}} = \frac{U_{\mathrm{N}}+U_{\mathrm{inorg}}-E_{\mathrm{N}}-M_{\mathrm{N}}}{U_{\mathrm{N}}+U_{\mathrm{inorg}}}, \quad (2)$$

where G_N is consistent with G_N of Supplementary equation 1. By solving Supplementary equation 2 for G_N, we obtain

$$G_{\mathrm{N}} = NUE(U_{\mathrm{N}} + U_{\mathrm{inorg}}). \quad (3)$$

Combining Supplementary equations 1 and 3, the mass balance of microbial N becomes,

$$\frac{\mathrm{d}B_{\mathrm{N}}(t)}{\mathrm{d}t} = NUE\left(U_{\mathrm{N}} + U_{\mathrm{inorg}}\right) - BD, \quad (4)$$

where N loss pathways are now embedded in the parameter NUE.

Supplementary References

1. Manzoni, S., Taylor, P., Richter, A., Porporato, A. & Ågren, G. I. Environmental and stoichiometric controls on microbial carbon-use efficiency in soils. *New Phytol.* **196,** 79-91 (2012).

2. Frankena, J., Vanverseveld, H. W. & Stouthamer, A. H. Substrate and energy costs of the production of exocellular enzymes by Bacillus-licheniformis. *Biotechnol. Bioeng.* **32,** 803-812 (1988).
